# *N*,*N*′,*N*′′-Tris(2-nitro­benz­yl)-2,2′,2′′-nitrilo­triethan­aminium trichloride 1.41-hydrate

**DOI:** 10.1107/S1600536809022399

**Published:** 2009-06-20

**Authors:** Perla Elizondo, Sylvain Bernès, Blanca Nájera, Francisco Góngora

**Affiliations:** a DEP Facultad de Ciencias Químicas, UANL, Guerrero y Progreso S/N, Col. Treviño, 64570 Monterrey, NL, Mexico

## Abstract

The title compound, C_27_H_36_N_7_O_6_^3+^·3Cl^−^1.41H_2_O, is the hydro­chloride of a tripodal amine, and was structurally characterized because the free base, used as a ligand in podate complexes, is an oily material. In the cation, the secondary amine groups are protonated, and, despite the induced Coulombic repulsions, a claw-like conformation is stabilized, with a cavity approximating *C*_3_ point symmetry. Such a topology, with the lone pair of the tertiary N atom placed inside the cavity, allows the encapsulation of guest species. Indeed, three chloride counter-ions balance the charges, one of which is located inside the cation cavity and is strongly bonded to the NH_2_^+^ groups. The asymmetric unit is completed by two water mol­ecules with occupancies 0.793 (11) and 0.621 (9). The crystal structure is formed by a complex network of efficient N—H⋯Cl and O—H⋯Cl hydrogen bonds. One nitro group also forms weak contacts with a water mol­ecule.

## Related literature

For related tripodal amine structures, see: Hossain *et al.* (2004 ▶); Coyle *et al.* (2006 ▶); McKee *et al.* (2006 ▶); Lakshminarayanan *et al.* (2007 ▶); For the role of electron-withdrawing groups in these mol­ecules, see: Bryantsev & Hay (2005 ▶).
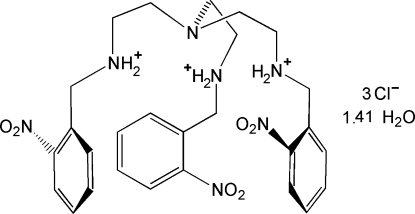


## Experimental

### 

#### Crystal data

C_27_H_36_N_7_O_6_^3+^·3Cl^−^·1.41H_2_O
*M* *_r_* = 686.47
Monoclinic, 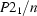

*a* = 9.131 (2) Å
*b* = 13.009 (4) Å
*c* = 28.071 (7) Å
β = 94.190 (9)°
*V* = 3326 (2) Å^3^
*Z* = 4
Mo *K*α radiation
μ = 0.33 mm^−1^
*T* = 298 K
0.50 × 0.42 × 0.24 mm

#### Data collection

Siemens P4 diffractometer
Absorption correction: ψ scan (*XSCANS*; Siemens, 1996 ▶) *T*_min_ = 0.819, *T*_max_ = 0.924
16115 measured reflections
6712 independent reflections
4181 reflections with *I* > 2σ(*I*)
*R*_int_ = 0.042
3 standard reflections every 97 reflections intensity decay: 3.5%

#### Refinement

*R*[*F* ^2^ > 2σ(*F* ^2^)] = 0.056
*wR*(*F* ^2^) = 0.175
*S* = 1.03
6712 reflections
438 parameters
12 restraints
H atoms treated by a mixture of independent and constrained refinement
Δρ_max_ = 0.34 e Å^−3^
Δρ_min_ = −0.34 e Å^−3^

### 

Data collection: *XSCANS* (Siemens, 1996 ▶); cell refinement: *XSCANS*; data reduction: *XSCANS*; program(s) used to solve structure: *SHELXS97* (Sheldrick, 2008 ▶); program(s) used to refine structure: *SHELXL97* (Sheldrick, 2008 ▶); molecular graphics: *SHELXTL* (Sheldrick, 2008 ▶) and *Mercury* (Macrae *et al.*, 2006 ▶); software used to prepare material for publication: *SHELXL97*.

## Supplementary Material

Crystal structure: contains datablocks I, global. DOI: 10.1107/S1600536809022399/im2103sup1.cif

Structure factors: contains datablocks I. DOI: 10.1107/S1600536809022399/im2103Isup2.hkl

Additional supplementary materials:  crystallographic information; 3D view; checkCIF report

## Figures and Tables

**Table 1:** Hydrogen-bond geometry (Å, °)

*D*—H⋯*A*	*D*—H	H⋯*A*	*D*⋯*A*	*D*—H⋯*A*
N2—H2*D*⋯Cl1	0.906 (10)	2.255 (11)	3.155 (2)	172 (3)
N12—H12*D*⋯Cl1	0.902 (10)	2.396 (19)	3.225 (3)	153 (3)
N22—H22*D*⋯Cl1	0.900 (10)	2.279 (11)	3.176 (3)	174 (3)
N12—H12*C*⋯Cl3	0.897 (10)	2.161 (11)	3.054 (3)	174 (3)
N22—H22*C*⋯Cl2	0.899 (10)	2.335 (14)	3.209 (3)	164 (3)
O8—H81⋯Cl1	0.850 (10)	2.35 (5)	3.115 (5)	150 (9)
O8—H82⋯Cl2	0.850 (10)	2.46 (4)	3.265 (5)	159 (10)
O7—H72⋯Cl3	0.851 (10)	2.28 (3)	3.102 (6)	163 (9)
N2—H2*C*⋯Cl2^i^	0.897 (10)	2.221 (14)	3.089 (3)	163 (3)
O7—H71⋯O6^ii^	0.851 (10)	2.45 (8)	2.965 (7)	119 (8)

Symmetry codes: (i) 
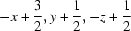
; (ii) 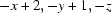.

**Fig. 1.**
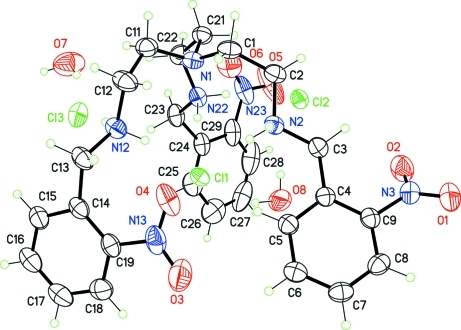
The asymmetric unit of (I). Displacement ellipsoids are shown at the 25% probability level.

**Fig. 2.**
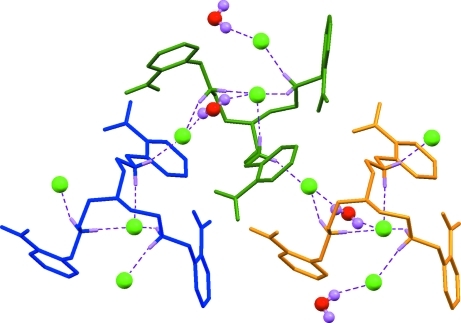
A part of the crystal structure of (I). Three symmetry-related cations are represented, omitting H atoms not involved in the supramolecular network. Hydrogen bonds are represented by dashed lines, and weak contacts involving nitro groups have been omitted for clarity. Symmetry codes for cations: green x, y, z; gold 3/2 - x, 1/2 + y, 1/2 - z; blue 3/2 - x, -1/2 + y, 1/2 - z.

## supplementary crystallographic information

### Comment

Tris(2-aminoethyl)amine (*tren*) derivatives are common starting materials for the synthesis of tripodal ligands used as podants in coordination chemistry (Coyle *et al.*, 2006). They are also used as anions receptors aiming at the design of molecules acting as selective anion bindig sites (Hossain *et al.*, 2004). On the other hand, for *tren*-based molecules bearing an aryl group, it has been shown that substitution of aryl with electron withdrawing groups enhances the stability of anions complexes (Bryantsev & Hay, 2005). Following this idea, we prepared tris[(2-nitrobenzylamino)ethyl]amine by reduction of the corresponding Schiff base, tris[(2-nitrobenzylideneamino)ethyl]amine, which had been previously characterized by X-ray diffraction (McKee *et al.*, 2006). However, as the free base is an oil, we transformed the amine into its hydrochloride salt, (I), and now report its X-ray structure.

The asymmetric unit (Fig. 1) contains one cation and three chloride ions balancing the charges, as expected. Two sites are occupied by water molecules, for which occupancies converged to 0.79 (11) and 0.621 (9). All atoms are placed in general positions. The presence of lattice water molecules is confirmed by IR spectroscopy, as well as by the consistent network of hydrogen bonds involving all H atoms of water molecules. The three secondary amine groups of the *tren* derivative are protonated, generating strong Coulombic repulsions within the cation. However, a claw-like conformation is stabilized, since a Cl^-^ ion is placed inside the cavity and forms three strong hydrogen bonds with all NH_2_^+^ groups. Such a behaviour is not systematically observed with closely related cations. For example, tris(2-benzylammonioethyl)amine cation has been crystallized with bromide or phosphate, and X-ray studies revealed that in both cases cations approximate a trigonal planar shape (Hossain *et al.*, 2004). In the same way, the free Schiff base used as starting material for (I) is a planar molecule with crystallographic *C*_3_ symmetry (space group *R*3, McKee *et al.*, 2006). In contrast, the same cation including pentafluorobenzyl groups in place of benzyl encapsulates Cl^-^ or Br^-^ ions (Lakshminarayanan *et al.*, 2007), as (I) does.

The supramolecular network formed by cations, anions, and water molecules in (I) is a complex arrangement of N—H···Cl and O—H···Cl hydrogen bonds. The most important, as commented above, are the NH_2_^+^···Cl1 strong hydrogen bonds allowing the anion encapsulation in the cationic cavity. Chloride ions placed outside this cavity are also connected to NH_2_^+^ functional groups *via* hydrogen bonds, one of which being intermolecular. In the asymmetric unit, water molecules also serve as donor for O—H···Cl hydrogen bonds (Fig. 2). One nitro group also forms weak intermolecular contacts with the water molecule O7.

### Experimental

Tris[(2-nitrobenzylideneamino)ethyl]amine (12.461 g, 27 mmol) was dispersed in methanol (40 ml). To achieve selective reduction, an amount of NaBH_4_ (3.643 g, 96 mmol) was added in small portions at 298 K under stirring. After reduction was completed, solvent was removed and then the reduction product, tris[(2-nitrobenzylamino)ethyl]amine, was extracted with CHCl_3_ (2 × 10 ml) and water (20 ml). The organic phase was dried over MgSO_4_. Evaporation of the solvent under reduced pressure afforded the free base as a pale yellow oil (yield: 91%). This compound was dissolved in ethanol and HCl was added until the title white salt [m.p. 503.5–504.5 K (dec.)] had completely precipitated. Suitable single crystals were obtained by evaporation of an ethanol-water (19:1) mixture.

### Refinement

From the IR spectrum of the single-crystal used for X-ray diffraction, it was assumed that an amount of water was present in the sample. Sites for disordered water molecules and chloride ions were inferred from H atoms positions, found in a difference map. Occupancies for water molecules were refined, and converged to 0.79 (1) and 0.621 (9) for O7 and O8. Some O atoms belonging to nitro groups display high displacement parameters, but attempts to resolve disordered sites were unsuccessful. N–bonded H atoms were found in a difference map, confirming the charge of the cation to be +3. All O– and N–bonded H atoms were refined freely, although the geometry was restrained to suitable target values: O—H = 0.85 (1) Å, H···H = 1.34 (2) Å, and N—H = 0.90 (1) Å. Other H atoms were placed in idealized positions and refined as riding to their parent atoms, with bond lengths fixed to 0.97 (methylene) or 0.93 Å (aromatic). Isotropic displacement parameters for H atoms were calculated as *U*_iso_(H) = 1.5 *U*_eq_(carrier atom) for water molecules and *U*_iso_(H) = 1.2 *U*_eq_(carrier atom) otherwise.

### Crystal data

**Table:**

C_27_H_36_N_7_O_6_^3+^·3Cl^−^·1.41H_2_O	*F*(000) = 1441
*M**_r_* = 686.47	*D*_x_ = 1.371 Mg m^−^^3^
Monoclinic, *P*2_1_/*n*	Melting point: 503.5–504.5 (dec.) K
Hall symbol: -P 2yn	Mo *K*α radiation, λ = 0.71073 Å
*a* = 9.131 (2) Å	Cell parameters from 69 reflections
*b* = 13.009 (4) Å	θ = 4.6–12.5°
*c* = 28.071 (7) Å	µ = 0.33 mm^−^^1^
β = 94.190 (9)°	*T* = 298 K
*V* = 3326 (2) Å^3^	Block, colorless
*Z* = 4	0.50 × 0.42 × 0.24 mm

### Data collection

**Table:**

Siemens P4 diffractometer	4181 reflections with *I* > 2σ(*I*)
Radiation source: fine-focus sealed tube	*R*_int_ = 0.042
graphite	θ_max_ = 26.3°, θ_min_ = 2.1°
2θ/ω scans	*h* = −11→10
Absorption correction: ψ scan (*XSCANS*; Siemens, 1996)	*k* = −16→1
*T*_min_ = 0.819, *T*_max_ = 0.924	*l* = −34→34
16115 measured reflections	3 standard reflections every 97 reflections
6712 independent reflections	intensity decay: 3.5%

### Refinement

**Table:**

Refinement on *F*^2^	Primary atom site location: structure-invariant direct methods
Least-squares matrix: full	Secondary atom site location: difference Fourier map
*R*[*F*^2^ > 2σ(*F*^2^)] = 0.056	Hydrogen site location: inferred from neighbouring sites
*wR*(*F*^2^) = 0.175	H atoms treated by a mixture of independent and constrained refinement
*S* = 1.03	*w* = 1/[σ^2^(*F*_o_^2^) + (0.0754*P*)^2^ + 1.5076*P*] where *P* = (*F*_o_^2^ + 2*F*_c_^2^)/3
6712 reflections	(Δ/σ)_max_ < 0.001
438 parameters	Δρ_max_ = 0.34 e Å^−^^3^
12 restraints	Δρ_min_ = −0.34 e Å^−^^3^
0 constraints	

### Fractional atomic coordinates and isotropic or equivalent isotropic displacement parameters (Å^2^)

**Table:**

	*x*	*y*	*z*	*U*_iso_*/*U*_eq_	Occ. (<1)
N1	0.9829 (3)	0.79136 (19)	0.17125 (9)	0.0624 (6)	
C1	0.9926 (4)	0.8169 (3)	0.22199 (11)	0.0701 (8)	
H1A	0.9977	0.8911	0.2253	0.084*	
H1B	1.0831	0.7887	0.2368	0.084*	
C2	0.8671 (4)	0.7782 (3)	0.24840 (11)	0.0690 (8)	
H2A	0.8467	0.7074	0.2393	0.083*	
H2B	0.8945	0.7797	0.2824	0.083*	
N2	0.7313 (3)	0.8412 (2)	0.23819 (8)	0.0586 (6)	
H2C	0.758 (3)	0.9045 (12)	0.2481 (11)	0.070*	
H2D	0.706 (3)	0.844 (2)	0.2064 (4)	0.070*	
C3	0.6071 (4)	0.7981 (2)	0.26312 (11)	0.0668 (8)	
H3A	0.6416	0.7807	0.2956	0.080*	
H3B	0.5740	0.7352	0.2472	0.080*	
C4	0.4792 (3)	0.8706 (2)	0.26437 (10)	0.0588 (7)	
C5	0.4586 (4)	0.9520 (2)	0.23318 (11)	0.0682 (8)	
H5A	0.5256	0.9624	0.2102	0.082*	
C6	0.3408 (4)	1.0185 (3)	0.23509 (13)	0.0733 (9)	
H6A	0.3284	1.0714	0.2129	0.088*	
C7	0.2432 (4)	1.0077 (3)	0.26895 (13)	0.0736 (9)	
H7A	0.1651	1.0533	0.2702	0.088*	
C8	0.2608 (4)	0.9293 (3)	0.30119 (12)	0.0725 (9)	
H8A	0.1957	0.9216	0.3249	0.087*	
C9	0.3756 (4)	0.8620 (2)	0.29820 (10)	0.0623 (8)	
N3	0.3838 (4)	0.7755 (3)	0.33158 (12)	0.0856 (9)	
O1	0.3524 (4)	0.7895 (3)	0.37214 (12)	0.1437 (14)	
O2	0.4250 (4)	0.6941 (2)	0.31824 (11)	0.1107 (10)	
C11	1.0831 (4)	0.8554 (3)	0.14559 (14)	0.0763 (9)	
H11A	1.0975	0.8234	0.1151	0.092*	
H11B	1.1777	0.8573	0.1637	0.092*	
C12	1.0314 (4)	0.9638 (3)	0.13682 (15)	0.0869 (11)	
H12A	1.0114	0.9950	0.1670	0.104*	
H12B	1.1091	1.0030	0.1235	0.104*	
N12	0.8964 (3)	0.9694 (2)	0.10352 (11)	0.0709 (7)	
H12C	0.907 (4)	0.923 (2)	0.0803 (9)	0.085*	
H12D	0.825 (3)	0.943 (3)	0.1206 (11)	0.085*	
C13	0.8715 (4)	1.0771 (3)	0.08618 (13)	0.0800 (10)	
H13A	0.9484	1.0955	0.0657	0.096*	
H13B	0.8785	1.1233	0.1134	0.096*	
C14	0.7249 (4)	1.0919 (2)	0.05908 (11)	0.0671 (8)	
C15	0.7256 (4)	1.0997 (3)	0.01013 (12)	0.0784 (10)	
H15A	0.8139	1.0911	−0.0039	0.094*	
C16	0.6024 (6)	1.1193 (3)	−0.01819 (15)	0.0926 (12)	
H16A	0.6072	1.1236	−0.0511	0.111*	
C17	0.4729 (5)	1.1328 (3)	0.00097 (16)	0.0919 (12)	
H17A	0.3891	1.1470	−0.0188	0.110*	
C18	0.4635 (4)	1.1258 (3)	0.04915 (17)	0.0858 (11)	
H18A	0.3737	1.1342	0.0623	0.103*	
C19	0.5905 (4)	1.1060 (2)	0.07840 (12)	0.0712 (9)	
N13	0.5780 (6)	1.1031 (2)	0.12976 (14)	0.0969 (11)	
O3	0.4647 (6)	1.1270 (4)	0.14496 (16)	0.1638 (18)	
O4	0.6834 (5)	1.0783 (2)	0.15623 (10)	0.1092 (10)	
C21	1.0212 (4)	0.6829 (2)	0.16548 (11)	0.0679 (8)	
H21A	0.9807	0.6430	0.1906	0.081*	
H21B	1.1272	0.6756	0.1689	0.081*	
C22	0.9657 (4)	0.6407 (3)	0.11834 (13)	0.0758 (9)	
H22A	0.9985	0.6840	0.0931	0.091*	
H22B	1.0057	0.5724	0.1144	0.091*	
N22	0.8036 (3)	0.63543 (19)	0.11434 (9)	0.0648 (7)	
H22C	0.774 (4)	0.596 (2)	0.1381 (9)	0.078*	
H22D	0.756 (3)	0.6949 (15)	0.1190 (12)	0.078*	
C23	0.7439 (4)	0.5962 (3)	0.06626 (11)	0.0769 (10)	
H23A	0.7383	0.6529	0.0437	0.092*	
H23B	0.8114	0.5457	0.0549	0.092*	
C24	0.5961 (5)	0.5488 (3)	0.06750 (11)	0.0737 (9)	
C25	0.4793 (6)	0.6015 (4)	0.04688 (14)	0.0959 (12)	
H25A	0.4936	0.6668	0.0345	0.115*	
C26	0.3370 (7)	0.5587 (6)	0.04397 (18)	0.1311 (19)	
H26A	0.2571	0.5950	0.0301	0.157*	
C27	0.3195 (8)	0.4606 (7)	0.0625 (2)	0.138 (3)	
H27A	0.2270	0.4303	0.0608	0.166*	
C28	0.4359 (10)	0.4094 (5)	0.0827 (2)	0.140 (3)	
H28A	0.4229	0.3436	0.0946	0.168*	
C29	0.5702 (6)	0.4512 (3)	0.08602 (12)	0.0939 (13)	
N23	0.6930 (7)	0.3884 (2)	0.10924 (12)	0.1083 (15)	
O5	0.6572 (7)	0.3081 (3)	0.12602 (13)	0.205 (3)	
O6	0.8142 (6)	0.4235 (3)	0.11446 (16)	0.1383 (14)	
Cl1	0.61515 (8)	0.83642 (6)	0.12955 (2)	0.0618 (2)	
Cl2	0.66871 (10)	0.53719 (6)	0.20643 (3)	0.0696 (2)	
Cl3	0.91772 (12)	0.82403 (9)	0.01891 (4)	0.0971 (3)	
O7	1.0384 (7)	0.6272 (5)	−0.0268 (2)	0.166 (3)	0.793 (11)
H71	1.046 (14)	0.658 (7)	−0.0533 (19)	0.249*	0.793 (11)
H72	1.006 (12)	0.673 (5)	−0.009 (3)	0.249*	0.793 (11)
O8	0.3919 (5)	0.6773 (5)	0.1646 (2)	0.121 (3)	0.621 (9)
H81	0.426 (9)	0.723 (5)	0.147 (3)	0.182*	0.621 (9)
H82	0.461 (7)	0.633 (6)	0.168 (4)	0.182*	0.621 (9)

### Atomic displacement parameters (Å^2^)

**Table:**

	*U*^11^	*U*^22^	*U*^33^	*U*^12^	*U*^13^	*U*^23^
N1	0.0575 (14)	0.0559 (14)	0.0721 (15)	0.0051 (12)	−0.0083 (12)	−0.0021 (12)
C1	0.0607 (19)	0.0697 (19)	0.077 (2)	0.0053 (16)	−0.0152 (16)	−0.0118 (16)
C2	0.070 (2)	0.071 (2)	0.0636 (18)	0.0112 (17)	−0.0087 (15)	−0.0050 (15)
N2	0.0642 (15)	0.0621 (14)	0.0480 (12)	0.0035 (12)	−0.0052 (11)	−0.0073 (11)
C3	0.076 (2)	0.0637 (18)	0.0605 (17)	0.0026 (16)	0.0023 (15)	0.0007 (14)
C4	0.0616 (18)	0.0628 (17)	0.0509 (15)	−0.0004 (14)	−0.0026 (13)	−0.0095 (13)
C5	0.072 (2)	0.0675 (19)	0.0659 (18)	0.0044 (17)	0.0064 (15)	0.0024 (15)
C6	0.073 (2)	0.0644 (19)	0.081 (2)	0.0048 (17)	−0.0035 (18)	0.0015 (16)
C7	0.0618 (19)	0.073 (2)	0.084 (2)	0.0042 (17)	−0.0072 (18)	−0.0149 (18)
C8	0.0571 (19)	0.088 (2)	0.073 (2)	−0.0054 (18)	0.0035 (15)	−0.0145 (18)
C9	0.0652 (19)	0.0674 (18)	0.0530 (16)	−0.0069 (16)	−0.0055 (14)	−0.0046 (14)
N3	0.087 (2)	0.096 (2)	0.075 (2)	0.0044 (19)	0.0149 (16)	0.0109 (18)
O1	0.163 (3)	0.182 (4)	0.091 (2)	0.048 (3)	0.042 (2)	0.042 (2)
O2	0.139 (3)	0.0850 (19)	0.109 (2)	0.0062 (19)	0.0188 (19)	0.0254 (17)
C11	0.060 (2)	0.075 (2)	0.092 (2)	−0.0038 (17)	−0.0063 (17)	0.0100 (18)
C12	0.078 (2)	0.072 (2)	0.105 (3)	−0.0145 (18)	−0.030 (2)	0.0173 (19)
N12	0.0734 (18)	0.0586 (16)	0.0781 (19)	−0.0116 (14)	−0.0118 (15)	0.0082 (13)
C13	0.085 (2)	0.066 (2)	0.084 (2)	−0.0186 (18)	−0.0207 (18)	0.0212 (17)
C14	0.077 (2)	0.0526 (17)	0.0688 (19)	−0.0149 (16)	−0.0133 (16)	0.0096 (14)
C15	0.087 (2)	0.076 (2)	0.071 (2)	−0.0080 (19)	−0.0006 (18)	0.0149 (17)
C16	0.115 (4)	0.082 (3)	0.077 (2)	−0.007 (2)	−0.020 (2)	0.0171 (19)
C17	0.099 (3)	0.070 (2)	0.101 (3)	−0.009 (2)	−0.035 (3)	0.016 (2)
C18	0.078 (2)	0.0571 (19)	0.123 (3)	−0.0040 (18)	0.007 (2)	0.013 (2)
C19	0.095 (3)	0.0479 (16)	0.0705 (19)	−0.0080 (16)	0.0032 (18)	0.0081 (14)
N13	0.143 (4)	0.0622 (18)	0.088 (2)	−0.004 (2)	0.030 (3)	0.0144 (17)
O3	0.183 (4)	0.170 (4)	0.150 (3)	0.037 (3)	0.090 (3)	0.048 (3)
O4	0.182 (3)	0.0783 (18)	0.0662 (16)	−0.005 (2)	−0.0007 (18)	0.0081 (13)
C21	0.0662 (19)	0.0653 (19)	0.0718 (19)	0.0132 (16)	0.0023 (15)	0.0041 (15)
C22	0.080 (2)	0.067 (2)	0.082 (2)	0.0100 (17)	0.0165 (18)	−0.0071 (17)
N22	0.088 (2)	0.0529 (14)	0.0541 (14)	0.0044 (13)	0.0074 (13)	−0.0057 (11)
C23	0.110 (3)	0.066 (2)	0.0553 (17)	−0.0139 (19)	0.0089 (17)	−0.0046 (15)
C24	0.110 (3)	0.0599 (18)	0.0524 (17)	−0.0175 (19)	0.0113 (18)	−0.0092 (14)
C25	0.123 (4)	0.096 (3)	0.068 (2)	−0.014 (3)	0.001 (2)	−0.013 (2)
C26	0.125 (5)	0.181 (6)	0.086 (3)	−0.011 (4)	−0.003 (3)	−0.042 (4)
C27	0.138 (5)	0.185 (7)	0.098 (4)	−0.088 (5)	0.044 (4)	−0.053 (4)
C28	0.207 (7)	0.133 (5)	0.087 (3)	−0.087 (5)	0.055 (4)	−0.043 (3)
C29	0.152 (4)	0.077 (2)	0.0545 (19)	−0.039 (3)	0.022 (2)	−0.0207 (18)
N23	0.213 (5)	0.0461 (18)	0.0650 (18)	−0.013 (3)	0.006 (3)	−0.0040 (14)
O5	0.416 (8)	0.075 (2)	0.113 (3)	−0.073 (3)	−0.056 (4)	0.0271 (19)
O6	0.187 (4)	0.079 (2)	0.151 (3)	0.034 (3)	0.029 (3)	0.007 (2)
Cl1	0.0686 (5)	0.0594 (4)	0.0555 (4)	0.0021 (4)	−0.0085 (3)	0.0005 (3)
Cl2	0.0815 (5)	0.0606 (4)	0.0665 (5)	0.0027 (4)	0.0050 (4)	0.0078 (3)
Cl3	0.1002 (7)	0.1081 (8)	0.0858 (6)	−0.0133 (6)	0.0264 (5)	0.0007 (5)
O7	0.139 (5)	0.212 (6)	0.145 (5)	0.047 (4)	0.003 (4)	−0.046 (4)
O8	0.077 (3)	0.125 (5)	0.163 (5)	−0.006 (3)	0.025 (3)	0.052 (4)

### Geometric parameters (Å, °)

**Table:**

N1—C1	1.459 (4)	C14—C19	1.389 (5)
N1—C11	1.465 (4)	C15—C16	1.354 (5)
N1—C21	1.466 (4)	C15—H15A	0.9300
C1—C2	1.497 (5)	C16—C17	1.346 (6)
C1—H1A	0.9700	C16—H16A	0.9300
C1—H1B	0.9700	C17—C18	1.364 (6)
C2—N2	1.497 (4)	C17—H17A	0.9300
C2—H2A	0.9700	C18—C19	1.396 (5)
C2—H2B	0.9700	C18—H18A	0.9300
N2—C3	1.485 (4)	C19—N13	1.455 (5)
N2—H2C	0.897 (10)	N13—O3	1.190 (5)
N2—H2D	0.906 (10)	N13—O4	1.216 (5)
C3—C4	1.504 (4)	C21—C22	1.487 (5)
C3—H3A	0.9700	C21—H21A	0.9700
C3—H3B	0.9700	C21—H21B	0.9700
C4—C5	1.378 (4)	C22—N22	1.478 (5)
C4—C9	1.393 (4)	C22—H22A	0.9700
C5—C6	1.385 (5)	C22—H22B	0.9700
C5—H5A	0.9300	N22—C23	1.507 (4)
C6—C7	1.357 (5)	N22—H22C	0.899 (10)
C6—H6A	0.9300	N22—H22D	0.900 (10)
C7—C8	1.365 (5)	C23—C24	1.487 (5)
C7—H7A	0.9300	C23—H23A	0.9700
C8—C9	1.372 (5)	C23—H23B	0.9700
C8—H8A	0.9300	C24—C25	1.361 (6)
C9—N3	1.463 (4)	C24—C29	1.398 (5)
N3—O2	1.193 (4)	C25—C26	1.411 (7)
N3—O1	1.208 (4)	C25—H25A	0.9300
C11—C12	1.501 (5)	C26—C27	1.391 (9)
C11—H11A	0.9700	C26—H26A	0.9300
C11—H11B	0.9700	C27—C28	1.345 (9)
C12—N12	1.493 (4)	C27—H27A	0.9300
C12—H12A	0.9700	C28—C29	1.338 (8)
C12—H12B	0.9700	C28—H28A	0.9300
N12—C13	1.495 (4)	C29—N23	1.498 (7)
N12—H12C	0.897 (10)	N23—O6	1.196 (6)
N12—H12D	0.902 (10)	N23—O5	1.202 (5)
C13—C14	1.503 (5)	O7—H71	0.851 (10)
C13—H13A	0.9700	O7—H72	0.851 (10)
C13—H13B	0.9700	O8—H81	0.850 (10)
C14—C15	1.378 (5)	O8—H82	0.850 (10)
			
C1—N1—C11	110.8 (3)	C14—C13—H13B	108.9
C1—N1—C21	109.2 (2)	H13A—C13—H13B	107.8
C11—N1—C21	109.3 (3)	C15—C14—C19	116.6 (3)
N1—C1—C2	114.4 (3)	C15—C14—C13	116.4 (3)
N1—C1—H1A	108.7	C19—C14—C13	126.8 (3)
C2—C1—H1A	108.7	C16—C15—C14	122.3 (4)
N1—C1—H1B	108.7	C16—C15—H15A	118.8
C2—C1—H1B	108.7	C14—C15—H15A	118.8
H1A—C1—H1B	107.6	C17—C16—C15	120.5 (4)
N2—C2—C1	112.0 (3)	C17—C16—H16A	119.8
N2—C2—H2A	109.2	C15—C16—H16A	119.8
C1—C2—H2A	109.2	C16—C17—C18	120.5 (4)
N2—C2—H2B	109.2	C16—C17—H17A	119.7
C1—C2—H2B	109.2	C18—C17—H17A	119.7
H2A—C2—H2B	107.9	C17—C18—C19	119.1 (4)
C3—N2—C2	110.7 (2)	C17—C18—H18A	120.5
C3—N2—H2C	114 (2)	C19—C18—H18A	120.5
C2—N2—H2C	104 (2)	C14—C19—C18	121.0 (3)
C3—N2—H2D	109 (2)	C14—C19—N13	121.3 (4)
C2—N2—H2D	111 (2)	C18—C19—N13	117.8 (4)
H2C—N2—H2D	109 (3)	O3—N13—O4	121.4 (4)
N2—C3—C4	113.3 (3)	O3—N13—C19	118.8 (5)
N2—C3—H3A	108.9	O4—N13—C19	119.8 (4)
C4—C3—H3A	108.9	N1—C21—C22	112.6 (3)
N2—C3—H3B	108.9	N1—C21—H21A	109.1
C4—C3—H3B	108.9	C22—C21—H21A	109.1
H3A—C3—H3B	107.7	N1—C21—H21B	109.1
C5—C4—C9	115.3 (3)	C22—C21—H21B	109.1
C5—C4—C3	122.5 (3)	H21A—C21—H21B	107.8
C9—C4—C3	122.2 (3)	N22—C22—C21	111.1 (3)
C4—C5—C6	121.7 (3)	N22—C22—H22A	109.4
C4—C5—H5A	119.1	C21—C22—H22A	109.4
C6—C5—H5A	119.1	N22—C22—H22B	109.4
C7—C6—C5	120.9 (3)	C21—C22—H22B	109.4
C7—C6—H6A	119.5	H22A—C22—H22B	108.0
C5—C6—H6A	119.5	C22—N22—C23	112.2 (3)
C6—C7—C8	119.4 (3)	C22—N22—H22C	109 (2)
C6—C7—H7A	120.3	C23—N22—H22C	111 (2)
C8—C7—H7A	120.3	C22—N22—H22D	116 (2)
C7—C8—C9	119.3 (3)	C23—N22—H22D	106 (2)
C7—C8—H8A	120.4	H22C—N22—H22D	102 (3)
C9—C8—H8A	120.4	C24—C23—N22	112.9 (3)
C8—C9—C4	123.4 (3)	C24—C23—H23A	109.0
C8—C9—N3	117.1 (3)	N22—C23—H23A	109.0
C4—C9—N3	119.5 (3)	C24—C23—H23B	109.0
O2—N3—O1	122.1 (4)	N22—C23—H23B	109.0
O2—N3—C9	119.0 (3)	H23A—C23—H23B	107.8
O1—N3—C9	118.9 (4)	C25—C24—C29	117.9 (4)
N1—C11—C12	114.5 (3)	C25—C24—C23	117.7 (3)
N1—C11—H11A	108.6	C29—C24—C23	124.4 (4)
C12—C11—H11A	108.6	C24—C25—C26	121.0 (5)
N1—C11—H11B	108.6	C24—C25—H25A	119.5
C12—C11—H11B	108.6	C26—C25—H25A	119.5
H11A—C11—H11B	107.6	C27—C26—C25	118.1 (6)
N12—C12—C11	112.6 (3)	C27—C26—H26A	121.0
N12—C12—H12A	109.1	C25—C26—H26A	121.0
C11—C12—H12A	109.1	C28—C27—C26	120.2 (6)
N12—C12—H12B	109.1	C28—C27—H27A	119.9
C11—C12—H12B	109.1	C26—C27—H27A	119.9
H12A—C12—H12B	107.8	C29—C28—C27	121.3 (6)
C12—N12—C13	110.5 (2)	C29—C28—H28A	119.4
C12—N12—H12C	107 (2)	C27—C28—H28A	119.4
C13—N12—H12C	114 (2)	C28—C29—C24	121.5 (6)
C12—N12—H12D	104 (2)	C28—C29—N23	117.5 (5)
C13—N12—H12D	115 (2)	C24—C29—N23	121.0 (4)
H12C—N12—H12D	104 (3)	O6—N23—O5	124.0 (6)
N12—C13—C14	113.2 (3)	O6—N23—C29	120.1 (4)
N12—C13—H13A	108.9	O5—N23—C29	115.4 (6)
C14—C13—H13A	108.9	H71—O7—H72	104 (3)
N12—C13—H13B	108.9	H81—O8—H82	104 (3)
			
C11—N1—C1—C2	163.5 (3)	C16—C17—C18—C19	0.9 (6)
C21—N1—C1—C2	−76.1 (3)	C15—C14—C19—C18	0.7 (5)
N1—C1—C2—N2	−75.8 (3)	C13—C14—C19—C18	175.6 (3)
C1—C2—N2—C3	177.4 (2)	C15—C14—C19—N13	−177.8 (3)
C2—N2—C3—C4	165.9 (2)	C13—C14—C19—N13	−2.9 (5)
N2—C3—C4—C5	19.9 (4)	C17—C18—C19—C14	−1.0 (5)
N2—C3—C4—C9	−158.0 (3)	C17—C18—C19—N13	177.6 (3)
C9—C4—C5—C6	−1.4 (4)	C14—C19—N13—O3	171.6 (4)
C3—C4—C5—C6	−179.5 (3)	C18—C19—N13—O3	−6.9 (5)
C4—C5—C6—C7	2.0 (5)	C14—C19—N13—O4	−7.1 (5)
C5—C6—C7—C8	−0.8 (5)	C18—C19—N13—O4	174.3 (3)
C6—C7—C8—C9	−1.0 (5)	C1—N1—C21—C22	159.9 (3)
C7—C8—C9—C4	1.7 (5)	C11—N1—C21—C22	−78.8 (3)
C7—C8—C9—N3	−176.1 (3)	N1—C21—C22—N22	−67.4 (4)
C5—C4—C9—C8	−0.5 (4)	C21—C22—N22—C23	178.1 (3)
C3—C4—C9—C8	177.6 (3)	C22—N22—C23—C24	155.5 (3)
C5—C4—C9—N3	177.2 (3)	N22—C23—C24—C25	107.9 (4)
C3—C4—C9—N3	−4.7 (4)	N22—C23—C24—C29	−75.9 (4)
C8—C9—N3—O2	143.3 (4)	C29—C24—C25—C26	−0.5 (5)
C4—C9—N3—O2	−34.6 (5)	C23—C24—C25—C26	176.0 (3)
C8—C9—N3—O1	−38.7 (5)	C24—C25—C26—C27	−0.5 (6)
C4—C9—N3—O1	143.5 (4)	C25—C26—C27—C28	0.4 (8)
C1—N1—C11—C12	−76.1 (3)	C26—C27—C28—C29	0.6 (8)
C21—N1—C11—C12	163.6 (3)	C27—C28—C29—C24	−1.7 (7)
N1—C11—C12—N12	−66.1 (4)	C27—C28—C29—N23	179.9 (5)
C11—C12—N12—C13	−165.5 (3)	C25—C24—C29—C28	1.6 (5)
C12—N12—C13—C14	−170.5 (3)	C23—C24—C29—C28	−174.6 (4)
N12—C13—C14—C15	−104.5 (4)	C25—C24—C29—N23	180.0 (3)
N12—C13—C14—C19	80.5 (4)	C23—C24—C29—N23	3.8 (5)
C19—C14—C15—C16	−0.4 (5)	C28—C29—N23—O6	−177.6 (4)
C13—C14—C15—C16	−175.9 (3)	C24—C29—N23—O6	3.9 (6)
C14—C15—C16—C17	0.4 (6)	C28—C29—N23—O5	−5.1 (5)
C15—C16—C17—C18	−0.6 (6)	C24—C29—N23—O5	176.4 (4)

### Hydrogen-bond geometry (Å, °)

**Table:**

*D*—H···*A*	*D*—H	H···*A*	*D*···*A*	*D*—H···*A*
N2—H2D···Cl1	0.91 (1)	2.26 (1)	3.155 (2)	172 (3)
N12—H12D···Cl1	0.90 (1)	2.40 (2)	3.225 (3)	153 (3)
N22—H22D···Cl1	0.90 (1)	2.28 (1)	3.176 (3)	174 (3)
N12—H12C···Cl3	0.90 (1)	2.16 (1)	3.054 (3)	174 (3)
N22—H22C···Cl2	0.90 (1)	2.34 (1)	3.209 (3)	164 (3)
O8—H81···Cl1	0.85 (1)	2.35 (5)	3.115 (5)	150 (9)
O8—H82···Cl2	0.85 (1)	2.46 (4)	3.265 (5)	159 (10)
O7—H72···Cl3	0.85 (1)	2.28 (3)	3.102 (6)	163 (9)
N2—H2C···Cl2^i^	0.90 (1)	2.22 (1)	3.089 (3)	163 (3)
O7—H71···O6^ii^	0.85 (1)	2.45 (8)	2.965 (7)	119 (8)

Symmetry codes: (i) −*x*+3/2, *y*+1/2, −*z*+1/2; (ii) −*x*+2, −*y*+1, −*z*.
